# Epidemiology of chronic kidney diseases in the Republic of Guinea; future dialysis needs

**DOI:** 10.12860/jnp.2015.24

**Published:** 2015-10-01

**Authors:** Alpha Oumar Bah, Cisse Lamine, Mamadou Cellou Balde, Mamadou Lamine Yaya Bah, Lionel Rostaing

**Affiliations:** ^1^Nephrology Unit, Donka National Hospital, Conakry, Republic of Guinea; ^2^Department of Nephrology and Organ Transplantation, CHU Rangueil, TSA 50032, Université Paul Sabatier, Toulouse, France; ^3^INSERM U563, IFR–BMT, CHU Purpan, Toulouse, France

**Keywords:** Chronic kidney diseases, End-stage renal disease, Hemodialysis

## Abstract

*Background:* Chronic kidney disease (CKD) is increasing worldwide and can lead to end-stage renal disease (ESRD).

*Objectives:* Because few patients with ESRD in the Republic of Guinea have access to haemodialysis, we retrospectively evaluated the prevalence of CKD, ESRD and access to supportive therapies.

*Patients and Methods:* 579 CKD patients (304 males; mean age: 44 ± 16 years) were admitted into Conakry nephrology department, the only centre in the Republic of Guinea, between 2009 and 2013. Most patients (63%) resided within Conakry (the capital), 12.5% came from lower Guinea, 11.7% from middle Guinea, 7.9% from upper Guinea and 4.8% from forest Guinea.

*Results:* Reasons for referral were increased serum creatinine (49.5%), hypertension (27%) and diffuse edema (17%). Also, 11% were diabetic, 12.5% were smokers, 17% were HIV-positive, 8.3% were HBV-positive and 15% were HCV-positive. The most frequent symptom at admission was nausea/vomiting (56%). Upon admission, 70.5% of patients already had ESRD. Although no kidney biopsies were performed it was assumed that 34% and 27% of patients had vascular nephropathy and chronic glomerulonephritis, respectively. Of the 385 ESRD patients, only 140 (36.3%) had access to haemodialysis (two sessions/week, 4 hours each). Most patients that received haemodialysis resided within the Conakry region (*P* < 0.0001). There were significant associations between mortality and (*i*) terminal stage of CKD (P = 0.0005), (*ii*) vascular nephropathy (*P* = 0.002), and (*iii*) nephropathies of unknown origin (*P* = 0.0001).

*Conclusions:* A fourfold increase in haemodialysis machines is needed in Conakry, plus four new nephrology/haemodialysis centres within the Republic of Guinea, each holding ≥30 haemodialysis machines.

**Implication for health policy/practice/research/medical education:**

This study shows that the prevalence of chronic kidney diseases (CKDs) in Republic of Guinea varies a lot from one area to the other. This is mainly due to the fact that there is a single nephrology center in the country: it is located in the capital, Conakry, which is excentrate. Therefore the referral to that center depends mainly on distance considerations and their related costs. In addition, when end-stage renal disease (ESRD) occurs the number of patients who are offered supportive therapy, i.e. dialysis depends on the place where they are living with regards to the distance to the single kidney center. This epidemiological study might help heath care authorities to establish the number of nephrology centers that might be opened in the country as well as the number of dialysis patients that might be treated.

## 1. Background

Chronic kidney disease (CKD) is an increasing major health problem worldwide. Its increased prevalence is mainly related to diabetes, hypertension and ageing populations in developed countries (1-4). However, in many developing countries, the major cause of CKD is related to infection-driven glomerulonephritis (5-8).

In developed countries when CKD is diagnosed, especially if at an early stage, treatments that halt the progress of CKD are implemented, e.g. antiproteinuria (angiotensin-converting enzyme inhibitors and/or angiotensin-receptor blockers) (9,10). These reduce impairment to renal function in many patients, thereby preventing or postponing the time until end-stage renal disease (ESRD) occurs and the need for supportive therapy, e.g. haemodialysis, peritoneal dialysis or kidney transplantation.

In developing countries, appropriate treatment of acute kidney failure, i.e. haemodialysis, is often efficacious as many patients recover with or without sequelae. However, there is a dilemma in developing countries that have increased rates of CKD and ESRD: should every ESRD patient be offered renal-replacement therapy or should this be only offered to young patients and/or to potential kidney-transplant recipients? Hence, implementing renal-replacement therapy in developing countries, i.e. mainly haemodialysis because of the logistical and hygiene issues with peritoneal dialysis and because kidney-transplantation programs take time to implement, creates huge costs that some countries cannot afford. In addition, the large proportion of health resources given to ESRD patients, who only make up a fraction of total medical cases, are then not available for other health issues, such as infectious diseases (e.g. HIV) or primary-care medicine (e.g. hypertension, diabetes, etc.). Finally, a key issue is that there are very few nephrologists in Africa (11).

In sub-Saharan countries, the epidemiology of CKD and ESRD is very different to that in developed countries (6,8,11). In the Republic of Guinea, CKD is mainly caused by infection-related nephropathies, hypertension and diabetes, and mostly affects young adults (12). CKD-related mortality is high because very few patients with ESRD have access to supportive therapies. In addition, CKD and ESRD can result in related social problems because some patients are no longer able to work. In addition, travel to undergo haemodialysis may be too far, necessitating patients to move to closer to the hospital, but they then lose their jobs. This can represent a huge social upheaval and additional costs.

## 2. Objectives

Based on these considerations, we undertook this retrospective study to assess the burden of CKD and ESRD in the Republic of Guinea and to aid the public-health authorities in implementing more supportive therapies for ESRD.

## 3. Patients and Methods

The Republic of Guinea has 10.65 million inhabitants and is divided into four large regions. Lower-Guinea has a population of 2.3 million (excluding the capital); the capital, Conakry, has 1.7 million inhabitants. The other three regions (Middle Guinea, Higher Guinea and Forest Guinea) have populations of 2.05, 2.65, and 1.95 inhabitants, respectively. They are also further from the capital and have poor road access.

Within the Republic of Guinea, there is a single department of nephrology, which is located in Donka national hospital in Conakry. The medical staff comprises 3 senior nephrologists, 12 nephrology residents and 3 senior internists. The department has 15 beds and an outpatient clinic where about 300 patients are looked after per month. In addition there is a haemodialysis facility equipped with 10 generators conducting three shifts per day, six days a week and where, at the moment, each ESRD patient undergoes only two haemodialysis sessions per week. There are no other nephrologists elsewhere in the country. Therefore, wherever a CKD patient lives he/she has go to Conakry to receive a CKD workup by a nephrologist.

This study was conducted in the department of nephrology at Donka national hospital between January 2009 and December 2013. We included all patients that had CKD, defined as having an estimated glomerular-filtration rate (eGFR; according to the MDRD equation) of ≤60 mL/min. We excluded patients admitted for acute renal failure. To identify the target study population, we reviewed, (*i*) the diagnostic charts of all patients admitted into our hospital, (*ii*) the medical files of all patients admitted in our department or seen in our outpatient clinic, and (*iii*) the medical files of all *de novo* patients starting haemodialysis.

We defined three categories of patient referral; (*i*) emergency admission, (*ii*) referral from a provincial or local hospital or a private clinic; and (*iii*) patients already followed-up in our nephrology clinic.

### 3.1. Ethical issues

The research followed the tenets of the Declaration of Helsinki. Informed consents were obtained. All patients took part in this study voluntary. The research was approved by ethical committee of Donka national hospital of Conakry, Republic of Guinea.

### 3.2. Statistical analysis

The retrospective data were collected by all of us and were entered into Excel (Microsoft Office 2010) software. Statistical analyses were performed using Epi Info 3.5.4 software. A *P* value of <0.05 was considered statistically significant.

## 4. Results

**Figure 1:**
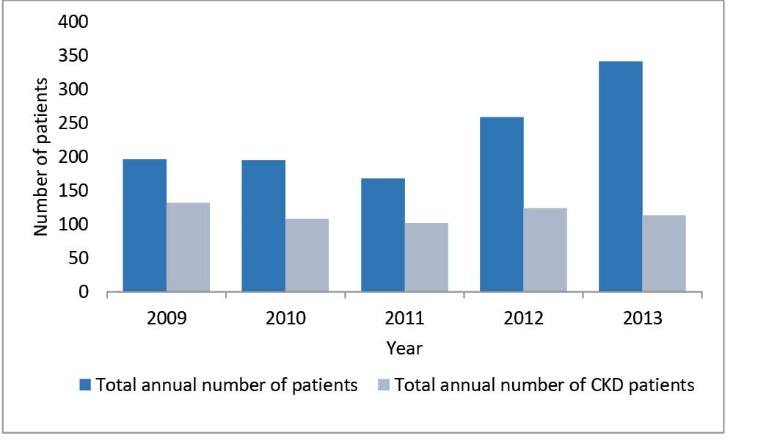
Annual incidence of chronic kidney disease (CKD). Prevalence amongst patients admitted for the first time into the department of nephrology at Donka national hospital, Conakry, Republic of Guinea.

**Figure 2:**
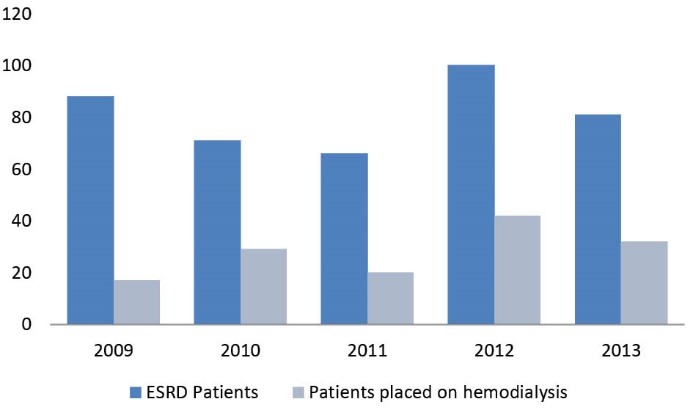
Annual incidence of end-stage renal disease patients that were offered haemodialysis.

**Figure 3:**
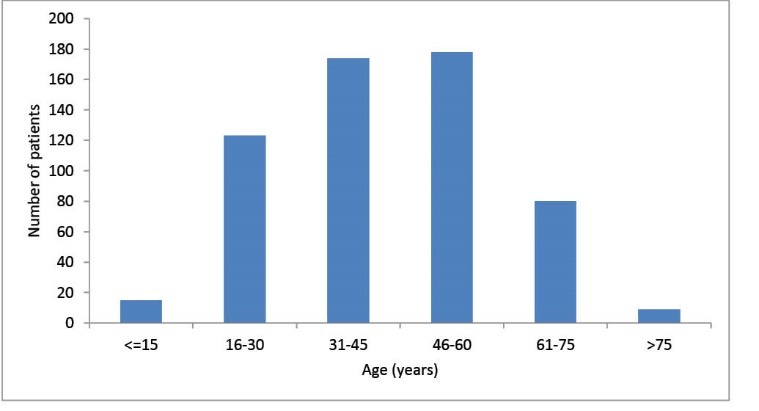
Prevalence of chronic kidney-disease patients according to their age at admission during the period 01-2009 to 12-2012.

During the period 2009-2012, 1161 patients were admitted into our nephrology department. Of these, 579 (49%) presented with CKD, and 304 (52%) were males. The mean age was 44 ± 16 years (range: 9-85 years). Figure 1 shows the annual incidence of CKD amongst patients admitted for the first time into our department. The data show relative stability with regards to new CKD patients, even though the annual number of patients seen in our department is sharply increasing over time. Figure 2 shows the repartition of the patients’ ages: most were aged between 31 and 60 years (60.7%), with the percentages of those aged between 31 and 45 similar to those aged 46-60 years (i.e. 30.0% and 30.7%, respectively). However, most patients (53.8%) were aged <45 years. With regards to the patients’ origins, Figure 3 shows that 63% were from Conakry and 12.5% from nearby Lower Guinea (12.5%).

Patients from the other three regions, which are much further from the capital, represented a total of only 24.4% of our CKD patients even though these three regions have a total population of 7 million, i.e. 63% of the whole country’s population. There was an inverse relationship between the distance from Conakry to a given region and the numbers of CKD patients admitted into our nephrology department, i.e. 11.7% were from Middle Guinea, 7.9% were from higher Guinea and 4.8% were from forest Guinea (Figure 3). With regards to the patients’ professions, 31.4% were housewives, 18.5% were manual workers, 17.6% were state employees, 17.0% were traders, 9.0% were students and 6.5% had other professions.

Of the 579 patients, 123 (21.2%) were admitted as emergency cases, 315 (54.4%) were referred from other hospitals or private clinics, and 141 (24.3%) were already being followed-up in our outpatient clinic. Symptoms on admission were nausea/vomiting (56%), physical asthenia (42%), dyspnea (38%), peripheral edema (37%), headache (30.5%), dizziness (28%), oligoanuria (25%), epigastralgia (19.5%), anorexia (19.5%) and tinnitus (19%).

Of the total patients, 61.6% were suffering from hypertension, 12.4% were smokers, 11% had diabetes, 10% were alcoholic, and 3.3% had a history of urinary-tract infection and/or uropathy. Also, 8.5% were taking nephrotoxic medications. With regards to viral infections, 16% were HIV (+), 15% were HCV (+) and 8.3% were HBsAg (+).

Of the new patients to our clinic, 70.5% had ESRD (eGFR <15 mL/min), 16% had severe CKD (eGFR 15-30 mL/min), 12% had moderate CKD (eGFR 30-60 mL/min) and 1.4% had mild CKD (eGFR >60 mL/min).

**Figure 4:**
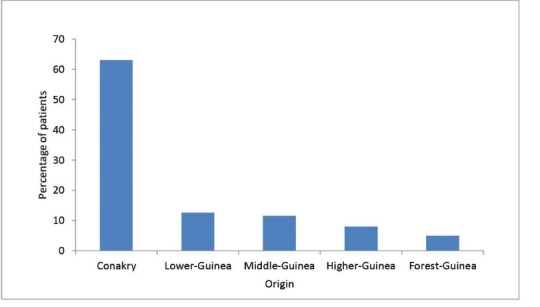
Chronic kidney-disease patients (n = 579) and their geographical origins during the period 01-2009 to 12-2012.

Because kidney biopsies are rarely performed in the Republic of Guinea, we could only refer to the patient’s medical history and/or biological parameters to assess the causes of CKD (Figure 4). One-third of cases of CKD were associated with a long history of hypertension (vascular nephropathy). In 26.7% of cases, proteinuria (with or without haematuria) suggested chronic glomerulonephritis. When diabetes was present or when HIV serology was positive, CKD was associated with these conditions. If there was a history of chronic urinary infection or uropathy, the patients were assumed to have interstitial nephropathy. There were very few hereditary nephropathies.

**Figure 5:**
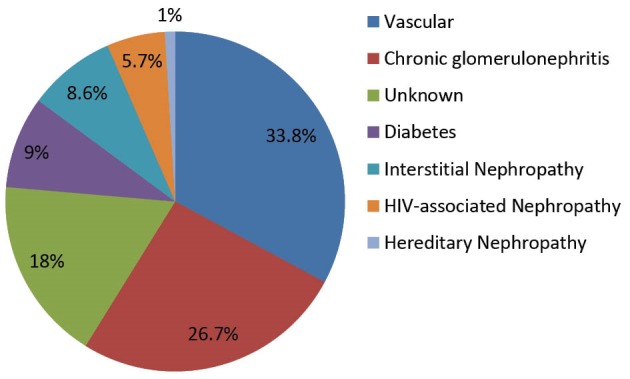
Potential causes of chronic kidney diseases in the 579 patients.

Figure 5 shows the annual number of ESRD patients that could be placed on chronic haemodialysis in our department for twice a week (4 hours each session). During the study period, <40% (140/385) of the ESRD patients received haemodialysis. There was also great variation with regards to where these patients lived: our study showed that of the 218 ESRD patients from Conakry area, 103 received haemodialysis compared to only 37 of the 167 patients that lived outside the Conakry area (*P *< 0.001).

Overall, 151 of the 579 patients (i.e. 26%) with CKD died within the study period. Most, i.e. 81% (n = 123) had ESRD and 16 (10%) were at stage 4. Conversely, mortality was very low for CKD stages 3 (10/60) and 2 (2/6) (*P *= 0.0005).We found that overall mortality was significantly associated with vascular nephropathies (36/160: 22%) (*P *= 0.002) and nephropathies of unknown origin (37/50: 74%) (*P *= 0.0001). Mortality was also significantly associated with ESRD (*P *= 0.0005).

For patients who were placed on haemodialysis, overall mortality was 38.6% (54 of the 140 patients).

## 5. Discussion

This retrospective study was performed in the only nephrology department within the Republic of Guinea. Our results show that access to supportive treatment in the setting of ESRD was significantly related to the distance between where the patients lived and the location of the nephrology centre. Thus, people with CKD that lived beyond Conakry or Lower Guinea (close to Conakry) had almost no chance of attending the nephrology facility. Even though only 24.4% of our patients originated from the three other regions in the country, these three regions represented 63% of the whole country’s population. Thus, there is serious and worrying discrimination with regards to these patients accessing specialized medical treatments, such as nephrology. This highlights the need for more nephrologists within the Republic of Guinea and the need to open at least one nephrology centre within each region. This could then supply effective haemodialysis facilities throughout the Republic of Guinea.

The Republic of Guinea is a medium-sized country (246 000 km²) in western Africa with a population of ~11 million. It has a very low developmental index, although it has many resources, such as bauxite, which is exported but does not add to the overall population’s wealth. In addition, within the last 2 years, the country has been hit by the Ebola virus, which has resulted in many deaths and a decline in the local economy (13,14).

Until very recently, there were no nephrologists or a nephrology department within the Republic of Guinea. At present, there are three nephrologists, who have all been trained in France. They all work at the single nephrology department at Donka national hospital. This department has 15 hospital beds, 10 haemodialysis machines and an outpatient clinic that welcomes ~300 patients per month. Patients either come alone or are referred by a GP or a private or public hospital located across the country. Outpatient clinic is almost free of charge as is the haemodialysis (3.2 US dollars per session), although the patient has to pay for hospitalization (~23 USD for a period not exceeding one month) and towards the cost of medications/perfusions.

In order to highlight the need to improve access and the care of CKD patients, we have conducted this retrospective study over the past 5 years. During this period, 579 CKD patients received treatment. Moreover, CKD was the leading cause for these patients’ hospitalization, i.e. CKD represented 50% of all hospitalizations. This figure is very similar to historical data, i.e. 52.5% (12) and represents a yearly average of 116 cases admitted for CKD. However, we are aware that this figure greatly underestimates the prevalence of CKD in the Republic of Guinea overall as many patients with CKD live outside the Conakry area: for example, patients that came from higher Guinea or forest Guinea represented only 8% and 5% of our cohort, respectively. This strongly suggests that patients who have CKD and live far from the capital have almost no chance of being referred to a nephrologist. In addition, wherever a patient lives, the costs of travel and of visiting a doctor are often too great for many patients.

The mean age of our CKD cohort was 44.4 years, and 82% were aged <60 years. This relatively young age for CKD patients is very similar to that found on the Ivory Coast,(15) Nigeria (16) and Congo(17) but differs greatly from that of American patients with CKD, where the mean age at diagnosis is 65 years (18). This is partly because life expectancy is significantly longer and because CKD is diagnosed much earlier in the US compared to sub-Saharan countries.

Most of our CKD patients were male, which has already been reported in previous studies; men have a 67% greater risk of developing CKD compared to women (19). In addition, housewives (31.5%) and manual workers (28%) were the most prevalent socioeconomic categories to have CKD, with similar data reported by Bah et al. (i.e. prevalence’s of 27.9% and 27.8%, respectively) (12). These high percentages may reflect that these categories are disadvantaged compared to middle-class persons or because they may have greater trust in traditional medicines.

With regards to risk factors, 61.6% of our patients had hypertension, 12.4% were active smokers and 11% were diabetic. These percentages may reflect changes to people’s behaviour in the Republic of Guinea over the last two decades, and why vascular nephropathies were the major cause of nephropathy in our cohort. This result is quite similar to that observed in North Africa (8) and South Africa (20,21).

With regards to symptoms, upon initial admission into our nephrology unit, gastrointestinal symptoms were the most common, i.e. nausea/vomiting (56%), followed by physical asthenia (42%) and dyspnoea (38%). This maybe because most of our patients presented with very late stage CKD, where uremic symptoms are predominant. This late referral may be partially because sick patients often first visit a local traditional doctor and then, only if their medical condition worsens and if they can afford it, do they consult a western-style doctor in Conakry.

HIV infection is quite prevalent in the Republic of Guinea: 1.7% of the general population tests positive. Within our CKD cohort, 17% were HIV (+), which seemed to have caused the CKD in 5.7% of cases. This raises the question of whether to test all CKD patients for HIV.

Markers for hepatitis B and C were present in 8.3% and 14.8% of our CKD patients, respectively. HBV infection can result in glomerulonephritis such as membranous nephropathy,(22) whereas chronic HCV infection may result in membranoproliferative glomerulonephritis, with or without cryoglobulinemia (23,24). Hence, as recommended by the KDIGO guidelines, renal abnormalities, such as proteinuria, haematuria and increased serum creatinine, should be searched for in HBV (+) and HCV (+) patients (25).

Because of the lack of primary-care medicine in the Republic of Guinea, in our series 70.5% of cases of CKD were found at a very advanced stage, when intervention was very unlikely to halt progress to renal failure. Vascular nephropathies and chronic glomerulonephritis accounted for 34% and 27% of nephropathies, respectively. Sabi et al (26), in Togo, found respective prevalences of 69% and 57.8%,respectively. Conversely, in France, vascular nephropathies (21%) and diabetes-related nephropathies (22%) represent almost half of the nephropathy cases (19). In our series, we could not classify the nephropathy in 18% of cases as most patients already had ESRD.

In our series, 66.5% of CKD patients presented with ESRD, and should have received dialysis. Of these, 218 (56.6%) were from the Conakry area. However, only 140 (36.4%) of the total 385 ESRD cases could be offered haemodialysis (two sessions per week). Of these, 103 (73.5%) were from the Conakry area whereas the other 37 were from other regions in the Republic of Guinea. Hence, patients with ESRD and not from the Conakry area had almost no chance of receiving haemodialysis when it was needed (*P *< 0.0001). This was mainly because patients from regions outside Conakry needed to stay in the capital for an indefinite period to attend haemodialysis sessions, which would have been too costly.

In the Republic of Guinea, 55% of the population lives in extreme poverty, i.e. <1.25 US dollars per day. This underlines the need for implementing haemodialysis facilities across the Republic of Guinea. In contrast, some very poor countries are able to offer haemodialysis to most of their patients, such as Madagascar, where 66% of ESRD patients are placed on haemodialysis (27).

Based on the current guidelines for treating ESRD patients in a developed country, patients should receive three sessions of haemodialysis a week, with each session lasting 4 hours. On a daily basis, three shifts can be performed (or even four) to maximize the utilization of dialysis machines. With this background it means that, for the Conakry area, the number of haemodialysis machines needs to be increased fourfold and that 30 haemodialysis machines need to be installed in each of four new centres located in other regions around the Republic of Guinea. It is almost certain that if nephrology centres are opened in other regions over the coming years, and if the population can be better screened for CKD, then the need for haemodialysis machines will also increase accordingly.

We also found a high mortality rate in our CKD patients. This has already been reported in some African series, such as in Nigeria (5). There was a statistically significant association between mortality and vascular nephropathies (*P *= 0.002) and nephropathies of unknown cause (*P *= 0.0001), and between mortality and the stage of CKD (*P *= 0.005). This high mortality rate may be partly associated with malnutrition, which commonly occurs with ESRD and other associated comorbidities.

## 6. Conclusions

In the Republic of Guinea, CKD mainly affects young adults who may also contribute to the work force; however, CKD is often only diagnosed at a very late stage, thus contributing to the high mortality rate. Moreover, because of a lack of financial and human resources (nurses, nephrologists), and access to dialysis machines, CKD-associated mortality has a huge negative impact on overall human quality-of-life and on the country’s economic development. These factors should be considered by politicians and health managers so that this dire situation can be remedied.

We urgently need a national program to improve the diagnosis of CKD earlier so that interventions may then halt the progress of this disease. The program requires the implementation of haemodialysis when necessary. In addition, because of the increased numbers of patients that need chronic haemodialysis, the authorities need to explore and develop kidney transplantation.

## 7. Limitations of the study

As we only took into account the patients that showed up in the single nephrology center of the country we certainly underestimated the actual prevalence of CKD in the country. Therefore, the needs are much more important as those we estimated.

## Authors’ contribution

BAO designed the study and analyzed the data. CL, BMC and BMLY collected the data and LR wrote the manuscript.

## Conflicts of interest

None.

## Funding/Support

None.
